# Supplementary material for: The Association of Ovarian Teratoma and Anti-N-Methyl-D-Aspartate Receptor Encephalitis: An Updated Integrative Review
Source: Int J Mol Sci. 2021 Oct 9;22(20):10911. doi: 10.3390/ijms222010911 (PMC8535897; doi:10.3390/ijms222010911)
Supplement: Supplementary file 1 [file ijms-22-10911-s001.zip › Table S1.pdf]

Table S1. The Modified Rankin Scale (mRS)	
The scale runs from 0–6, running from perfect health without symptoms to death.	
0	No symptoms
1	No significant disability. Able to carry out all usual activities, despite some symptoms
2	Slight disability. Able to look after own affairs without assistance, but unable to carry out all previous activities
3	Moderate disability. Requires some help, but able to walk unassisted
4	Moderately severe disability. Unable to attend to own bodily needs without assistance, and unable to walk unassisted
5	Severe disability. Requires constant nursing care and attention, bedridden, incontinent
6	Dead
