# Supplementary material for: The Association of Ovarian Teratoma and Anti-N-Methyl-D-Aspartate Receptor Encephalitis: An Updated Integrative Review
Source: Int J Mol Sci. 2021 Oct 9;22(20):10911. doi: 10.3390/ijms222010911 (PMC8535897; doi:10.3390/ijms222010911)
Supplement: Supplementary file 1 [file ijms-22-10911-s001.zip › Table S2.pdf]

**Table S2. Comparison of Neuroglial and Immune Cell Populations in ovarian teratomas with or without anti-NMDAR encephalitis [56]**			
**Characteristics**	**Teratomas with encephalitis (n=12)**	**Teratomas without encephalitis (n=61)**	**P value**
Immune cell population involving neuroglial tissue			
➢ Diffuse lymphoplasmacytic infiltrate in neuropil	30 (cells/hpf)	14 (cells/hpf)	< 0.05
➢ Presence of lymphoid aggregates without germinal centers around neuropil	100%	70%	< 0.05
➢ Presence of lymphoid aggregates without germinal centers around ganglia clusters	80%	11%	< 0.05
➢ Presence of lymphoid aggregates with germinal centers around neuropil	92%	7%	<0.0001
➢ Lymphoid aggregates with germinal centers around neuropil (number per tumor)	3	0	<0.0001
Immune cell population involving non-neuroglial tissue			
➢ Lymphoid aggregates without germinal centers (number per tumor)	3	10	< 0.01
Neuroglial population			
➢ Percent of teratoma composed of neuroglial tissues	5.2%	1.3%	< 0.01
➢ Astrocytes per hpf of neuropil	200	97	< 0.0001
➢ Mature neurons per hpf of neuropil	1	7	< 0.001
➢ NeuN positive cells per hpf of neuropil	0	18	< 0.01
➢ Total surface area of neuroglial tissues	0.1 cm^2	0.5 cm^2	< 0.001
Overall tumor size	1.9 cm	7.5 cm	< 0.0001
Hpf: high power field.			
